# The tetraspanin CD151 marks a unique population of activated human T cells

**DOI:** 10.1038/s41598-020-72719-8

**Published:** 2020-09-25

**Authors:** Mildred D. Perez, Lillian Seu, Kelsey E. Lowman, David C. Moylan, Christopher Tidwell, Shekwonya Samuel, Alexandra Duverger, Frederic H. Wagner, Eric Carlin, Vishal Sharma, Brandon Pope, Chander Raman, Nathan Erdmann, Jayme Locke, Hui Hu, Steffanie Sabbaj, Olaf Kutsch

**Affiliations:** 1 Department of Medicine, The University of Alabama at Birmingham, Birmingham, AL USA; 2 Department of Microbiology, The University of Alabama at Birmingham, Birmingham, AL USA; 3 Department of Surgery, The University of Alabama at Birmingham, Birmingham, AL USA

**Keywords:** Immunology, Inflammation, Lymphocytes

## Abstract

Tetraspanins are a family of proteins with an array of functions that are well studied in cancer biology, but their importance in immunology is underappreciated. Here we establish the tetraspanin CD151 as a unique marker of T-cell activation and, in extension, an indicator of elevated, systemic T-cell activity. Baseline CD151 expression found on a subset of T-cells was indicative of increased activation of the MAPK pathway. Following TCR/CD3 activation, CD151 expression was upregulated on the overall T-cell population, a quintessential feature of an activation marker. CD151+ T-cell frequencies in the spleen, an organ with increased immune activity, were twice as high as in paired peripheral blood samples. This CD151+ T-cell frequency increase was not paralleled by an increase of CD25 or CD38, demonstrating that CD151 expression is regulated independently of other T-cell activation markers. CD151+ T-cells were also more likely to express preformed granzyme B, suggesting that CD151+ T cells are pro-inflammatory. To this end, HIV-1 patients on antiretroviral therapy who are reported to exhibit chronically elevated levels of immune activity, had significantly higher CD4+CD151+ T-cell frequencies than healthy controls, raising the possibility that proinflammatory CD151+ T cells could contribute to the premature immunological aging phenotype observed in these patients.

## Introduction

Tetraspanins are a family of small proteins with four transmembrane-spanning domains that are involved in a series of cellular processes, including but not limited to the control of cell morphology, cell motility, apoptosis, and cell proliferation^1–6^. The role of tetraspanins in cancer development, aggressiveness and metastasis has been investigated in detail, but our understanding of the role of tetraspanins in immunology is still limited. The available studies, most of which were done in mouse models, indicate that tetraspanins regulate or at least modulate human T cell responses^7–11^. These modulating effects are primarily accomplished by the tetraspanin-coordinated formation of highly specialized tetraspanin-enriched microdomains (TEMs) also called tetraspanin-webs^12–14^. The formation of these poly-protein cell surface structures that can contain key T cell markers such as CD2, CD4, CD8, antigen presenting HLA molecules and integrins is the result of the ability of tetraspanins to laterally coordinate other proteins with extremely high efficiency. The ensuing spatial rearrangement of proteins into these TEMs then alters the activation state of the tetraspanin expressing cells, but some publications have found that tetraspanins themselves can directly signal^15–20^.

We previously expanded on these findings and provided evidence that in humans the tetraspanin CD151 acts as a marker of distinct T cell subpopulations. T cell expression frequencies were generally higher on CD8 T cells than on CD4 T cells, but on both T cell lineages, the CD151 frequency increased as a function of the memory differentiation state^21^. In vitro, CD151+ T cells proliferated in the absence of antigen or TCR/CD3 stimulation, driven by only IL-2, suggesting that CD151 T cells have a hyper-responsive phenotype. Proteomic analysis further revealed that CD151 in T cells actively changed the cell status, in particular by altering cell cycle control and cell death pathway functionalities. The observed hyper-responsive phenotype of CD151+ T cells is in line with reports that describe CD151 as a marker of tumor aggressiveness and the reported role of CD151 in TCR/CD3 signaling^22–31^.

We now provide evidence that CD151 is a sensitive T cell activation marker. Its baseline presence on T cells in the absence of TCR activation is indicative of elevated MAPK/ERK pathway activity, and accordingly, following TCR/CD3 engagement by either antigen or antibodies, CD151 is upregulated on both CD4 and CD8 T cells. Consistent with the idea that CD151 is an activation marker, CD151+ T cell frequencies were found significantly higher at sites of elevated immune activity (spleen). CD151+ T cell frequencies were also generally increased in pathological settings that are characterized by low-level immune hyperactivation as found in HIV-1 patients on antiretroviral therapy.

## Results

### CD151 expression on T cells is upregulated following TCR/CD3 activation

**Figure 1:**
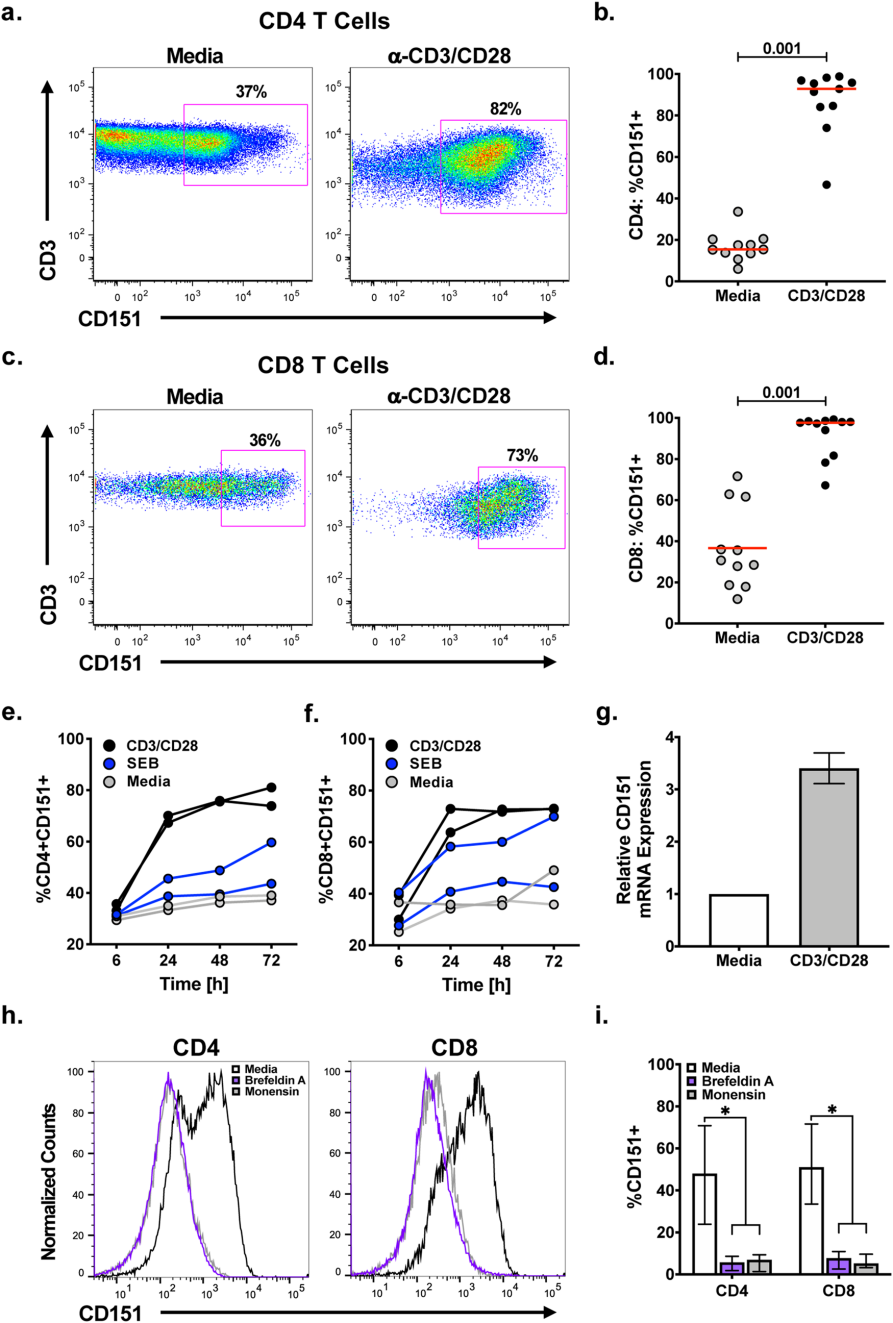
CD151 expression is upregulated on T cells following TCR activation. CD151 expression on CD4+ and CD8+ T cells in PBMCs was determined at baseline or following activation with an anti-CD3/CD28 mAb combination (OKT3/CD28.2) using flow cytometric analysis. Representative flow plots for (**a**) CD4+ and (**c**) CD8+ T cells showing the increase in the CD151 expression frequency following stimulation. The percentage of CD151+ (**b**) CD4+ and (**d**) CD8+ T cells in the peripheral blood of 11 healthy individuals at baseline (gray circles) and on day 4 following stimulation with an anti-CD3/CD28 mAb combination (black circles) presented as a beeswarm plot. The red lines represent the median values. PBMC from 2 healthy volunteers were stimulated with anti-CD3/CD28 mAb (black line) or with SEB (blue line), and CD151 expression was determined on (**e**) CD4+ T cells and (**f**) CD8+ T cells over a period of 72 h. CD151 expression on unstimulated cells is depicted by gray circles. (**g**) Twenty-four hours after anti-CD3/CD28 mAb stimulation, the increase in relative CD151 mRNA expression in CD4+ T cells was measured using qRT-PCR. (**h**) Representative histograms of PBMC stimulated with anti-CD3/CD28 mAb and treated with brefeldin A (purple line) or monensin (gray line) after 6 h to block cell surface transport of newly synthesized proteins. CD151 expression was then determined 24 h post activation. The black histograms represent stimulated cultures with no transport inhibitor treatment. (**i**) CD4+CD151 + and CD8+CD151+ T cell frequencies in PBMCs from six donors 24 h post anti-CD3/CD28 stimulation cultured in either the absence or presence of brefeldin A or monensin treatment.

We recently reported that a subpopulation of T cells in the peripheral blood constitutively expresses the tetraspanin CD151 and that these cells exhibit an increased propensity to proliferate in the absence of cognate antigen recognition^22^. This phenotype prompted us to address the question of whether CD151 would function as a T cell activation marker. To test this hypothesis, we stimulated human peripheral blood mononuclear cells (PBMCs) with an anti-CD3/CD28 mAb combination, an experimental equivalent of TCR-mediated recognition of cognate antigen, and monitored changes to the frequency of T cells expressing CD151 using flow cytometry. The representative flow cytometry plots in Fig. 1a,c show the increase in the percentage of CD151+ T cells 72 h post anti-CD3/CD28 mAb stimulation for CD4+ and CD8+ T cells. CD151 expression remained upregulated for at least 96 h post stimulation with the median percentage of CD4+CD151+T cells from 11 healthy donors increasing from 15% (6–34%) without stimulation to 93% (74–99%) (Fig. 1b) and the percentage of CD8+CD151+ T cells increasing from 31% (12–63%) to 98% (78–99%) (Fig. 1d). Kinetic analysis of the CD151 expression response to stimulation with an anti-CD3/CD28 mAb combination demonstrated that 24 h post activation, preceding any proliferative response, this increase in the CD151+ T cell frequency was almost complete in the CD4 T cell population (Fig. 1e) and had reached maximum levels in the CD8 T cell population (Fig. 1f).

Stimulation with SEB, also triggered upregulation of CD151 expression, albeit on a smaller percentage of T cells, which is consistent with the selective activity of SEB against only a subset of TCR Vβ chains. However, the SEB-induced increase of CD151 expression confirms that the signal required for CD151 induction is mediated through TCR engagement (Fig. 1e,f, blue lines). Induction of CD151 protein expression triggered by anti-CD3/CD28 mAb stimulation was reflective of a ~ 3.5-fold increase in CD151 mRNA expression (Fig. 1g). Stimulation experiments performed in the presence of protein transport inhibitors demonstrated the requirement of protein synthesis and transport for the increase in CD151 expression. Treatment of primary T cells with either brefeldin A, an inhibitor of protein transport from the ER to the Golgi, or monensin, which inhibits protein transport from the medial to the trans Golgi cisternae, blocked CD3/CD28 mAb-induced upregulation of CD151 on CD4+ and CD8+ T cells with > 80% efficiency (Fig. 1i). Together, these data demonstrate that TCR/CD3 pathway activation triggers the synthesis of CD151-specific mRNA and subsequent de novo protein synthesis with protein transport being essential for the observed CD151 upregulation.

### Proliferating T cells are characterized by high levels of CD151 expression

**Figure 2:**
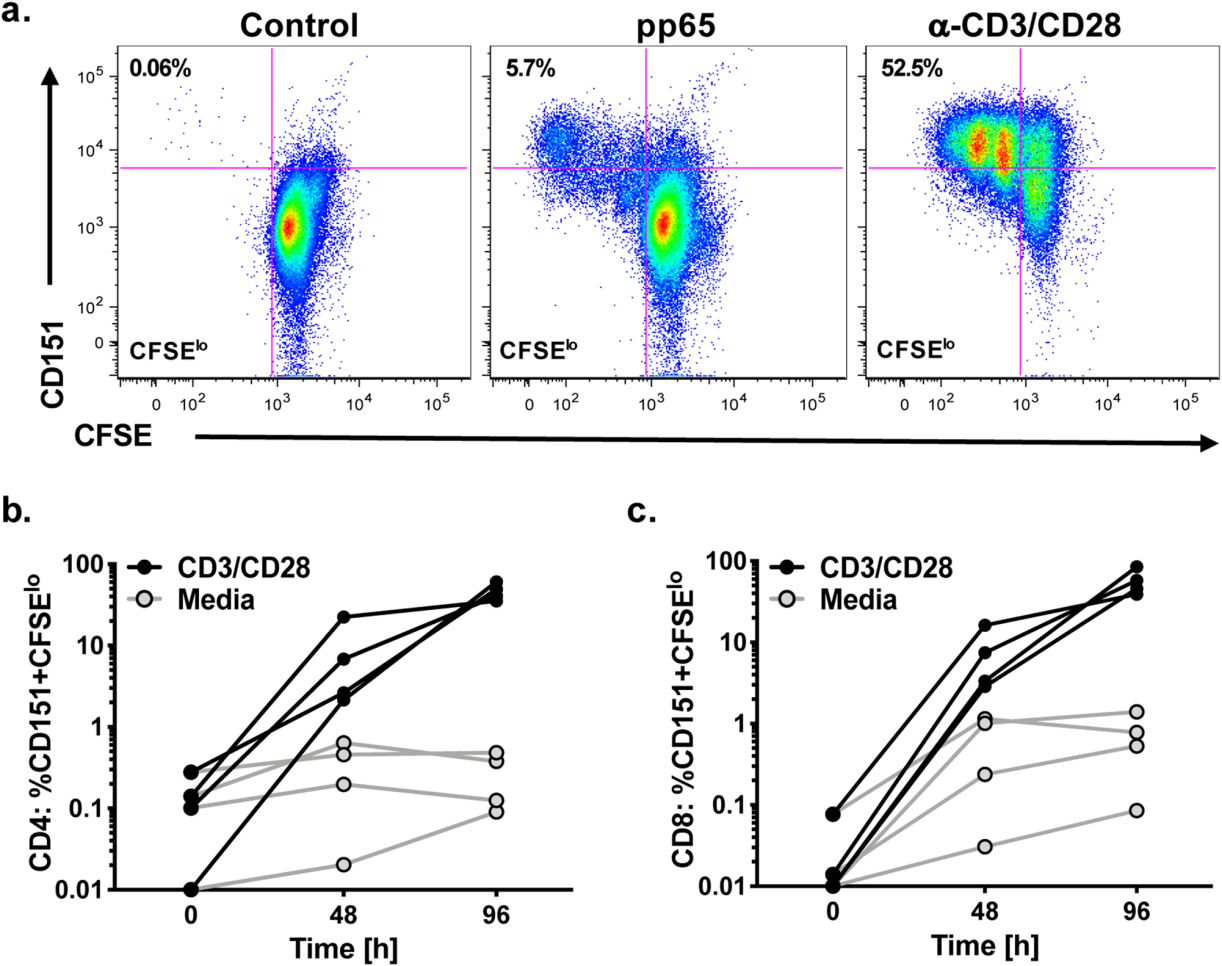
CD151 expression as a function of T cell proliferation. (**a**) CFSE-labeled PBMCs were stimulated with either a CMV pp65 antigen peptide library or anti-CD3/CD28 mAbs. Four days post stimulation, cell proliferation was determined as the reduction of the CFSE signal in relation to the regulation of CD151 expression using flow cytometric analysis. Flow plots show CD151 expression on proliferating CD4+ T cells (CFSE^lo^) and non-proliferating CD4+ T cells. The depicted experiment is representative for four tested donors. PBMC from four healthy individuals were stimulated with anti-CD3/CD28 mAb (black lines) or left unstimulated (media, gray lines) for various lengths of time and the expression of CD151 on proliferating cells (CFSE^lo^) was determined for (**b**) CD4+ T cells and (**c**) CD8+ T cells. Unstimulated cells served the negative controls.

We next investigated the relation of CD151 expression and cell proliferation following activation. For these experiments, we stained T cells with carboxyfluorescein succinimidyl ester (CFSE; proliferation) and stimulated the cells with anti-CD3/CD28 mAb or a CMV-pp65 peptide pool (Fig. 2a). As a negative control, no stimulus was added. After 4 days (peak proliferation) the cells were stained for CD151 expression. Flow plots for PBMCs from a representative healthy volunteer that also demonstrated responses to CMV-pp65 (used to determine if antigen-specific cells would mirror mitogenic stimulation) are shown. It can be noted that all proliferating cells also expressed CD151 regardless of the utilized stimulus (Fig. 2a). Proliferation data from 4 healthy volunteers indicated that the CD4+ T cells (Fig. 2b) and CD8+ T cells (Fig. 2c) that had undergone the most extensive proliferation, and were therefore CFSE^lo^, exhibited the highest CD151 expression levels. For comparison, unstimulated cells, used as a negative control (media, gray line), are shown.

### CD151 marks a unique subset of T cells

**Figure 3:**
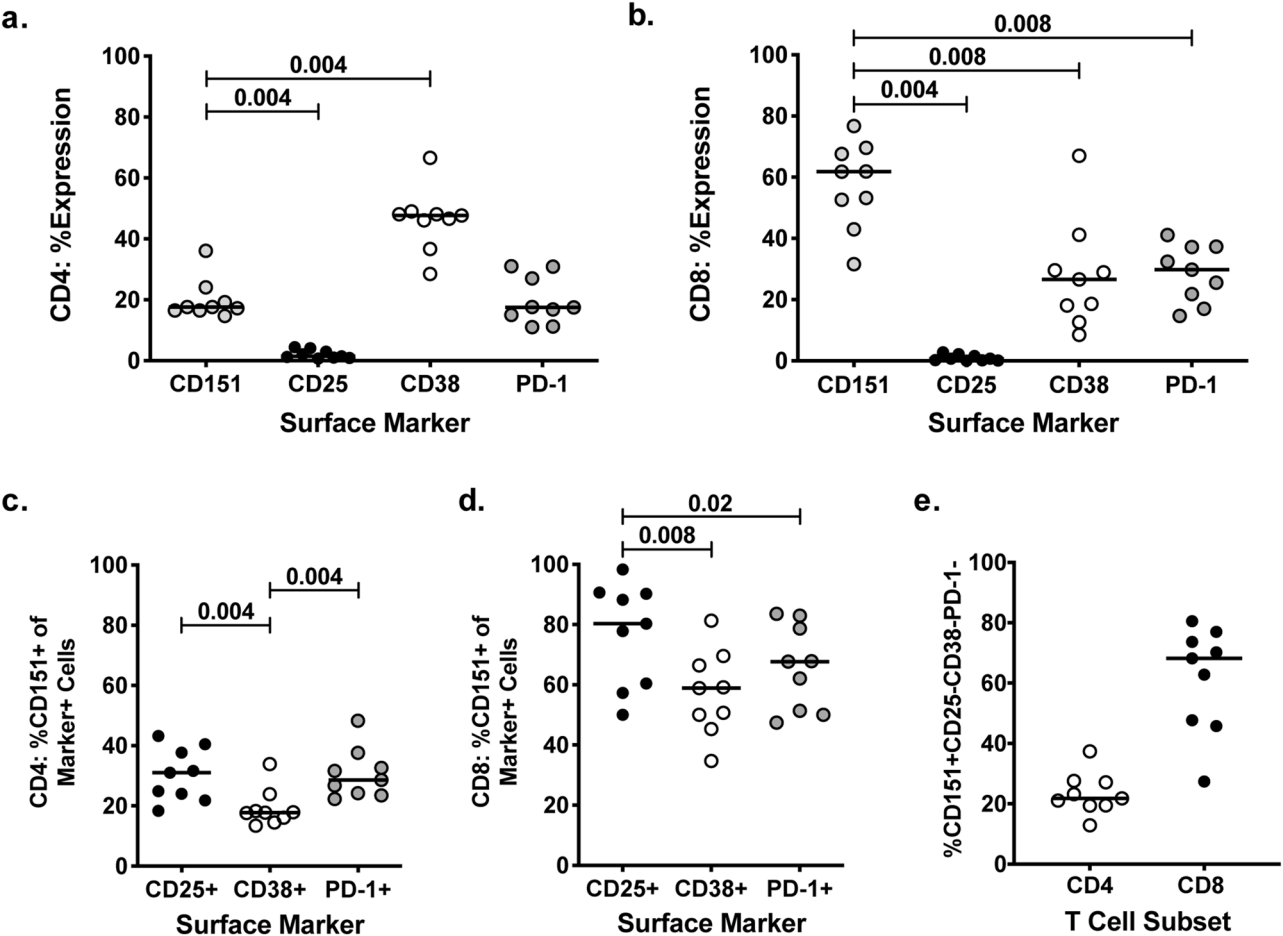
CD151 expression on CD4+ and CD8+ T cells in relation to established T cell activation markers. PBMC from 9 healthy individuals were co-stained for CD151 and the established T cell activation markers CD25, CD38 and PD-1. Beeswarm plots show the expression frequency of CD151, CD25, CD38 and PD-1 on (**a**) CD4+ T cells and (**b**) CD8+ T cells. Percentage of CD25+, CD38+ and PD1+ (**c**) CD4+ T cells and (**d**) CD8+ T cells that also expressed CD151. (**e**) The frequency CD4+ and CD8+ T cells that expressed CD151 and not CD25, CD38, or PD-1. Median values are shown as black lines.

Having shown that CD151 is a marker of T cell activation and proliferation, we next addressed whether CD151 would mark a distinct population of activated T cells that would not be identified by other established T cell activation markers, such as CD25^33,34^, CD38^35,36^, or PD-1, which is upregulated after T cell activation but also can act as an exhaustion marker^37,38^. For this purpose, we analyzed the possible association of CD151 expression with these markers on peripheral blood T cells from healthy donors using flow cytometric analysis. The results indicate that CD151 expression marks a distinct T cell population that is not described by either of the established T cell activation markers (Fig. 3). Ex vivo analysis of PBMCs from a total of 9 healthy donors revealed that a median of 18% of CD4+ T cells and 62% of CD8+ T cells expressed CD151, while only 1.4% of CD4 T cells and 0.7% of CD8 T cells expressed CD25. CD38 expression was observed on 48% of the CD4+ T cells, which is higher than the CD4+CD151+ T cell frequency, but only 27% of CD8+ T cells expressed CD38, which is lower than the CD8+CD151+T cell frequency. The median PD-1 expression frequency in the CD4+ T cell population (18%) was not statistically different from the median frequency of CD4+CD151+ T cells, however, PD-1 was expressed on significantly fewer CD8+ T cells (30%) than CD151 (62%) (Fig. 3a,b). It was immediately obvious from these data that the ex vivo expression frequency for the three established activation markers, CD25, CD38, and PD-1, in peripheral T cells differed from the frequency of CD151 expressing T cells, suggesting that CD151 is regulated independently of these established activation markers. This became even more evident as each T cell population that was marked by one of the three established T cell activation markers was further resolved into two populations by the expression of CD151. For example, throughout the 9 donors, of the CD4+CD25+T cell population, a median of 31% was CD151+, while 18% of the CD4+CD38+ T cell sub-population and 29% of the CD4+PD1+ T cells were CD151+ as well (Fig. 3c). Similarly, CD151 further resolved all of the CD8+ T cell populations that were marked by one of these classic T cell activation markers (Fig. 3d). Of the CD4+CD151+, 22% (median range 13–37%) were uniquely marked by CD151 and did not express either CD25, CD38 or PD-1, while 68% (median range 27–81%) of all CD8+CD151+ T cells were not expressing CD25, CD38 or PD-1 (Fig. 3e).

### Higher CD151+ T cell frequency in T cells isolated from human spleen

**Figure 4:**
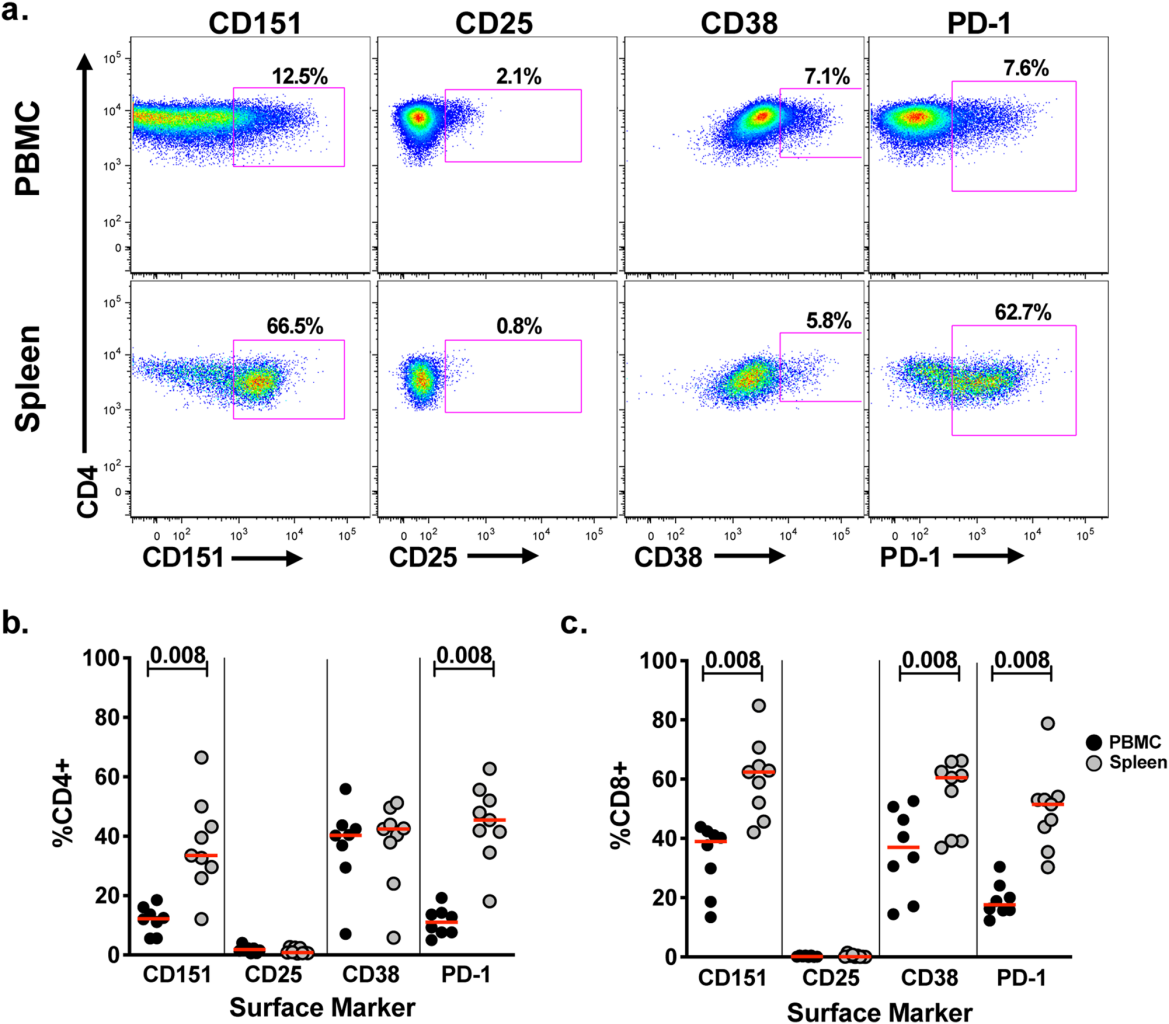
Increased CD151 expression frequency on human splenocytes. (**a**) PBMCs and matched splenocytes from 8 organ donors were analyzed for the expression of CD151, CD25, CD38 and PD-1 using flow cytometric analysis. Representative flow plots showing the expression of the various markers on CD3+CD4+ T cells from paired PBMCs and splenocytes. The frequency of CD151+, CD25+, CD38+ and PD1+ (**b**) CD4+ T cells and (**c**) CD8+ T cells in the peripheral blood and in splenocytes is depicted as a beeswarm plot. Median values are shown as red lines.

Different compartments of the immune system have been associated with differential levels of immune activity. While immune activity in the peripheral blood is considered generally low, secondary lymphoid organs, such as the spleen, are considered compartments with increased immune activity^39–47^. We reasoned that if CD151 functions as a sensitive marker of increased T cell baseline activity, we would find an elevated frequency of CD151+ T cells in this immunologic compartment. To probe this idea, we obtained paired PBMC and splenocyte material from 8 human donors. Ex vivo flow cytometric analysis of these samples revealed that CD151+ T cell frequencies would be associated with the immunological activation status of the immunologic compartment and were found increased in splenocytes when compared to peripheral blood. In a first step, we confirmed that T cell material from the peripheral blood of the organ donors would reproduce the results from blood samples of healthy donors. As seen in Fig. 4, this was indeed the case and the frequency ranges of CD151+, CD25+, CD38+ and PD1+ T cells isolated from peripheral blood of the organ donors matched those found in PBMCs from healthy donors.

Using flow cytometric analysis (Fig. 4a), we proceeded to compare CD151+ T cell frequencies between the matched peripheral blood/splenocytes pairs for CD4+ T cells (Fig. 4b) and CD8+ T cells (Fig. 4c). The median CD4+CD151+ T cell frequency in the peripheral blood was 12.3% (5.6–16.1%) compared to 33.5% (12.1–66.5%) in the spleen. For CD8+ T cells, the percentage of CD151+ cells increased from a median of 39% in the peripheral blood to 62.4% in the spleen. In neither compartment nor lineage did the percentage of CD25+ T cells, which in all cases stayed below 2%, parallel the increase of the CD151+ T cell frequency, indicating that in the absence of recent TCR/CD3 activation, CD151 expression is dissociated from the expression of CD25. The median frequency of CD4+CD38+ T cells did not change between the peripheral blood and spleen (PBMCs: 40.3%; spleen: 42.4%), however, splenocytes contained a higher percentage of CD8+CD38 T cells than PBMCs (PBMCs: 37.1%; spleen: 60.5%). The percentage of CD4+PD1+ T cells increased from 11.0% in the peripheral blood to 45.4% in the splenocyte population, which was a more pronounced increase than observed for CD4+CD151+ T cells. Similarly, a median of 17.6% of the T cells in the peripheral blood were CD8+PD1+ (lower % than CD8+CD151+) and this percentage increased in the spleen to 51.5%. By comparing the expression pattern of these activation markers in two separate compartments with reported differential immunological activity, we confirmed that CD151 is a T cell activation marker and that CD151 expression marks a distinct T cell population that is not described by either of the established T cell activation markers. In fact, as CD151 expression partially overlaps with each of these established T cell markers, future studies that include CD151 as a differential activation marker will allow more detailed studies of T cell activation at an even better resolution.

### The MAPK/ERK pathway is involved in the control of CD151 expression

As CD151 on peripheral blood T cells and T cells from the spleen, in the absence of in vitro stimulation, mostly marked T cells that did not express CD25, it stands to reason that the presence of CD151 indicates activation levels that are either insufficient to drive CD25 expression or that the presence of CD151 is indicative of an activated pathway that does not control CD25. To define the signal transduction pathways that would drive baseline CD151 expression and thus underlie the elevated baseline activation status of CD151+ T cells, we used antibody array-based kinome analysis data of sorted CD4+CD151+ in comparison to CD4+CD151− T cells. The utilized KAM-850 arrays detect 189 protein kinases, 31 protein phosphatases and 142 regulatory subunits of these enzymes and other cell signaling proteins. The array provides information on the phosphorylation state of 128 unique sites in protein kinases, 4 sites in protein phosphatases and 155 sites in other cell signaling proteins. Kinome data derived from sorted cells of 5 individuals indicated a substantial enrichment of increased signals for proteins involved in the MAPK/ERK signal transduction pathway^48,49^. Of the top 50 statistically significant increased kinome signals, 29 signals could be linked to this pathway (Table 1). Given the importance of the MAPK/ERK pathway for cell cycle control, these data suggest that the previously observed increased propensity of CD151+ T cells to proliferate without a requirement for cognate antigen recognition may be driven by an elevated baseline activation state of CD151+ T cells^22^. The increased signals throughout the PKC family, which are phosphorylation targets of MAPK/ERK are further consistent with previous reports that link CD151 with PKC activation^20,50,51^.

**Table 1:** Ranking of MAPK pathway associated proteins in top 50 proteins with altered activity/expression.

Rank	Protein	Phospho site	CD151-	CD151+	Fold regulation
1	eIF4E	S209	500	1531	3.06
2	PKCb1/2	T500	913	2490	2.73
3	RSK1/2	S363/S369	1208	2782	2.3
4	p38 MAPK14	Pan-specific	11425	26141	2.29
5	MEF-2	Pan-specific	5073	11199	2.21
6	Jun	Y170	672	1458	2.17
7	PKCd	Pan-specific	6982	13787	1.97
8	PKCa	S657	3917	7650	1.95
9	ERK1/2	Pan-specific	4402	8263	1.88
10	Btk	Pan-specific	10228	18418	1.8
11	ERK5	Pan-specific	20935	37538	1.79
14	PKCe	Pan-specific	4913	8314	1.69
17	MAPKAPK2	T334	1402	2354	1.68
18	Cyclin D1	Pan-specific	9954	16296	1.64
20	eIF2a	S52	3192	5218	1.63
21	TYK2	Pan-specific	27398	43928	1.6
22	ERK2	Pan-specific	20514	32595	1.59
24	PKCg	Pan-specific	9980	15512	1.55
25	p27 Kip1	Pan-specific	5231	8101	1.55
31	H2A.X	S139	894	1351	1.51
34	ERK5	T219+Y221	5661	8407	1.49
40	Hsp27	S78	13085	18808	1.44
41	4E-BP1	Pan-specific	770	1095	1.42
42	B-Raf	Pan-specific	7108	10093	1.42
43	MEK1	Pan-specific	28486	40417	1.42
44	NFKB p65	S529	820	1149	1.4
46	RSK1/2	S380/S386	1831	2549	1.39
48	Rb	Pan-specific	1261	1724	1.37
50	PKCd	S645	2709	3367	1.35

**Figure 5:**
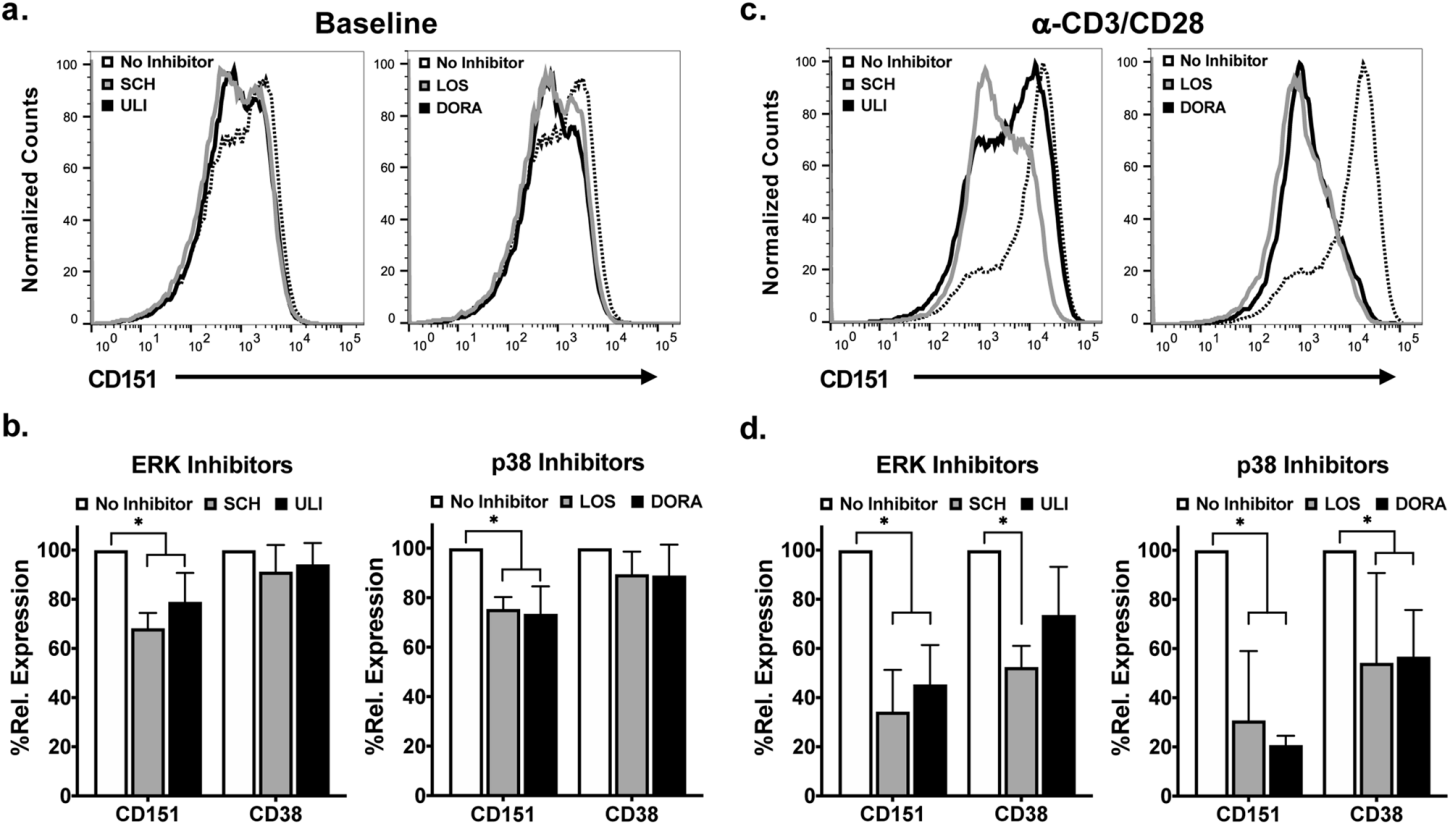
Effect of ERK and p38 pathway inhibition on CD151 expression in CD4+ T cells. PBMCs from four healthy individuals were treated with the ERK inhibitors (Ulixertinib, ULI or SCH772984, SCH), the p38 inhibitors (Losmapimod, LOS or Doramapimod, DORA), or DMSO as control, and 3 days post treatment analyzed for the expression of CD151 and CD38 using flow cytometric analysis. (**a**) Representative flow cytometry histogram plots showing the expression of CD151. (**b**) The effect of each inhibitor on baseline CD151 expression levels in comparison to the inhibitor effect on CD38 expression as determined for four individuals. (**c**) Representative flow cytometry histogram plots showing the expression of CD151 following anti-CD3/CD28 mAb stimulation in the absence or presence of the indicated inhibitors. (**d**) The effect of each inhibitor on activation-induced CD151 expression levels analyzed for the expression of CD151 and CD38 using flow cytometric analysis for CD4+ T cells. Due to extensive donor variation regarding CD151 expression levels (baseline MFI range 216–875) data are represented as relative MFI normalized for the CD151 baseline expression in untreated cells.

Guided by these results, we tested the effect of ERK and p38 inhibitors on CD151 baseline expression on primary CD4+ T cells (Fig. 5a,b). We chose Ulixertinib (BVD-523; ULI) and SCH900353 (MK-8353; SCH), which are both in clinical trials as ERK inhibitors, and Losmapimod (LOS) and Doramapimod (DORA; phase 3) as p38 inhibitors. Studying the effect of these inhibitors on CD151 expression on resting T cells is complicated by the low protein turnover and generally limited life expectancy of resting T cells in in vitro cell culture. To provide sufficient time for inhibitory effects to be reflected at the protein expression level, we thus determined CD151 expression levels on day 3 post addition of the inhibitors. We further determined the effect of the inhibitors on anti-CD3/CD28 mAb stimulation-induced CD151 expression.

As shown by representative histograms for CD151 expression derived from flow cytometric analysis, ERK inhibitors and p38 inhibitors decreased baseline CD151 expression on resting CD4 T cells (Fig. 5a). The inhibitory effect of each of these inhibitors on baseline CD151 expression in T cells was confirmed in experiments using PBMCs from 4 donors, and specificity was demonstrated by comparison with the inhibitor effects on baseline CD38 expression which was not significantly affected (CD4+ T cells: Fig. 5b; CD8+ T cells: Supplemental Figure S1). The importance of the MAPK pathway for CD151 expression control was confirmed by activation experiments (Fig. 5c,d), as all inhibitors reduced stimulation-induced CD151 expression in CD4 T cells and CD8 T cells to some extent (Supplemental Figure S1), but the p38 inhibitors Losmapimod and Doramapimod were more potent than the ERK inhibitors SCH900353 and Ulixertinib, the latter being a weaker inhibitor of CD151 upregulation (Fig. 5d). These data confirm the predictions derived from the kinome data on CD4+ T cells regarding the importance of the p38 MAPK pathway for the control of baseline CD151 expression and assign a role for the p38/ERK MAPK pathway in the control of activation-induced CD151 expression in T cells.

### CD151+ T cell frequencies are increased in pathological conditions associated with elevated immune activation

**Figure 6:**
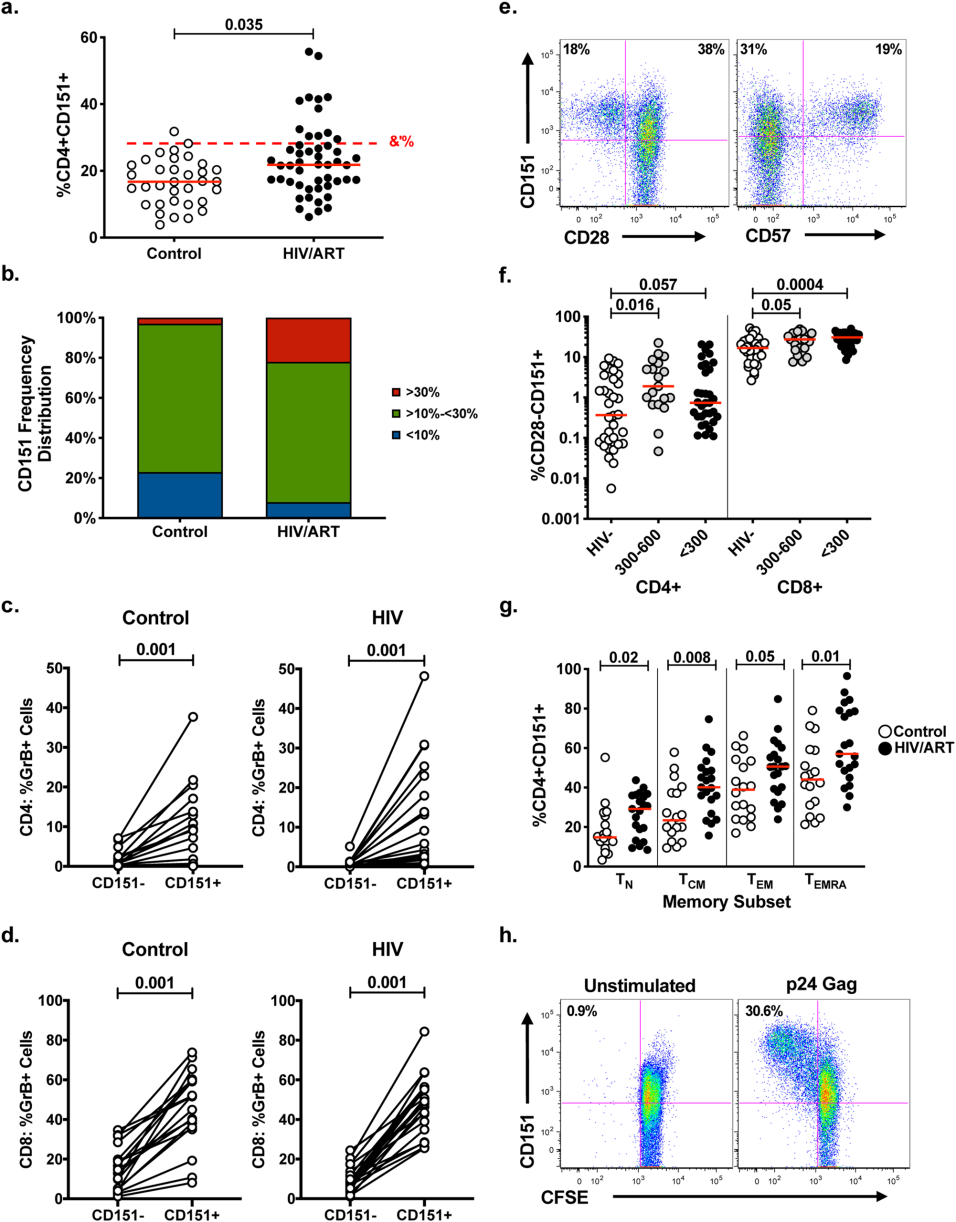
Increased CD4+CD151+ T cell frequencies in the peripheral blood of HIV-1 patients on fully suppressive ART. (**a**) Beeswarm plots describing the frequencies of CD4+CD151+ T cells from healthy donors (controls), and HIV-seropositive individuals on ART (HIV/ART). Red lines indicate median frequencies. The 95% interval for the normal distribution of CD151+ T cell frequencies in healthy donors (95% reference range) was calculated and is indicated as a red dotted line. (**b**) The bar graphs show the distribution of individuals based on their CD4+CD151+ T cell frequencies into groups with < 10% CD4+CD151+ T cells (blue), between 10%-30% (green) and > 30% (red), in healthy controls and HIV/ART patients. The frequency of CD151- and CD151+Cells in healthy and HIV/ART patients with preformed Granzyme B for (**c**) CD4+ T cells and (**d**) CD8+ T cells. (**e**) CD151+ T cell expression in relation to CD28 or CD57 expression on CD4+ T cells from HIV/ART patients. Numbers indicate the percentage of cells within each quadrant. (**f**) Relative contribution of the CD28− T cell population to the total CD151+ T cell population in healthy controls and HIV/ART patients with CD4+ T cells counts between 300 and 600 or < 300 cells/mm^3^. (**g**) Comparison of the frequency of CD4+CD151+ T cells as a function of the memory differentiation state between healthy control individuals (open symbols) and HIV/ART patients (closed symbols). T cell subsets were defined by patterns of CD45RA and CCR7 expression: Naïve T cells (T_N_: CD45RA+CCR7+), central memory T cells (T_CM_: CD45RA–CCR7+), effector memory T cells (T_EM_: CD45RA^–^ CCR7^–^) and T effector memory RA-positive cells (T_EMRA_: CD45RA+CCR7–). Median values are depicted by red lines. (**h**) CFSE-labeled PBMCs from a HIV-seropositive individual were stimulated with overlapping Gag peptides for 4 days and increased CD151 expression on proliferating Gag-specific T cells was quantified using flow cytometric analysis. Unstimulated cells served as the negative control.

Given that our data establish CD151 as a sensitive marker of increased baseline T cell activation, we tested whether the frequency of CD151+ T cells would be increased under pathological conditions that have been associated with elevated T cell activation. Immune hyperactivation has been described for HIV-1 patients despite long-term, successful suppression of viral replication by antiretroviral therapy (ART). This elevated immune activity has been correlated to an increased risk for inflammation-driven comorbidities including metabolic syndrome, diabetes, cardiovascular disease, neurodegenerative impairment, and cancer^52–62^. We proposed that the frequency of CD151+ T cells would be increased in HIV/ART patients if this cell compartment contributes to the reported hyperactive immune status. To test this hypothesis, we compared CD151+ T cell frequencies of a group of 52 HIV/ART patients, with the CD151+ T cell frequencies of individuals in a healthy control group (n = 33). As predicted, flow cytometric analysis revealed a statistically significant increase in the frequency of CD4+CD151+ T cells in HIV/ART patients relative to healthy controls (Fig. 6a) but not for CD8+ T cells (data not shown). At the same time, while the frequency of CD151+ T cells was increased, CD151 expression levels, as measured by the mean fluorescence intensity (MFI), on T cells from HIV/ART patients were not higher than on T cells obtained from healthy control donors (**Supplemental Figure S2).

While the data confirmed the hypothesis that CD151+ T cells are found more frequently under pathological conditions associated with increased immune activation, the increase in median frequency may be less interesting than the number of outliers among HIV/ART patients, patients that have CD4+CD151+ T cell counts that exceed the 95% reference range of normal CD4+CD151+ T cell counts of healthy individuals. As for other diagnostic reference ranges, a CD4+CD151+ T cell frequency range was calculated as the median CD4+CD151+ T cell frequency from 33 healthy (HIV-1 seronegative) individuals plus 2-times the standard deviation. The upper limit of the CD4+CD151+ T cell frequency reference range was determined to be 30% (Fig. 6a, %CD4+CD151 + position of red dotted line). By this definition, ~ 20% of HIV/ART patients had pathologically elevated CD4+CD151+ T cells counts (Fig. 6b, red bars). It is also noteworthy that only 5–10% of the HIV/ART patients had CD4+CD151+ T cell counts below 10%, compared to > 20% of the healthy donors (Fig. 6b, blue bar). The idea that CD4+CD151+ T cells could be pro-inflammatory based on a senescence-like phenotype and contribute to an inflammatory environment is suggested by the finding that CD151+ T cells are generally more likely to express preformed granzyme B than CD151− T cells. We found this to be the case in both CD4 (Fig. 6c) and CD8 T cells (Fig. 6d) for healthy controls and HIV/ART patients.

If the expression of CD151 is linked to hyperproliferation, we would expect to find CD151 expressed on senescent T cells that arise following extensive proliferation, generally marked by the loss of CD28 or the gain of CD57, which are more abundant in HIV/ART patients. Indeed, as demonstrated by flow cytometric analysis on ex-vivo (non-stimulated) CD4+ T cells from HIV/ART patients, CD151 was found expressed on almost all CD28-negative or CD57-positive cells (Fig. 6e). While the number of CD4+CD28−CD151+ T cells was increased in HIV/ART patients relative to healthy donors, CD4+CD28−CD151+ T cells still only accounted for a portion of the increase of the total CD4+CD151+ T cell population in HIV/ART patients (Fig. 6f).

The presence of CD151 on non-senescent CD28+ T cells could be possibly explained by a parallel observation found in another established T cell driven pathological condition. In rheumatoid arthritis, also a chronic inflammatory disease that has been associated with premature immunological aging^63,64^, activation was reported to be most pronounced in the naïve T cell population^65^. We thus proposed that a similar scenario would be found in HIV/ART patients and determined CD151 expression in the CD4+ T cell population from HIV/ART patients as a function of their memory differentiation status. As seen in Fig. 6g, a relative increase in the CD4+CD151+ T cell frequencies was seen throughout all memory T cell compartments, including the T_naive_ compartment. With naïve T cells constituting the largest T cell compartment, this means that the absolute increase in the activated CD4+CD151+ T cell population observed in HIV/ART patients, as for rheumatoid arthritis patients, is mostly driven out of the naïve T cell compartment.

While the mechanism(s) leading to the increased frequency of CD4+CD151+ T cells in HIV/ART patients so far remains undetermined, we find that HIV-specific CD4+ T cells that responded to p24 Gag antigen also upregulated CD151 expression (Fig. 6h), allowing for the possibility that parts of the increase of CD4+CD151+ T cells may reflect ongoing viral replication in immunologic reservoirs such as the gut associated lymphoid tissue that have been described^66^.

## Discussion

Beyond markers defining T cell lineages, subpopulations and differentiation status, an ever-increasing number of proteins have been identified that define T cell states (e.g. loss of CD28, gain of PD-1 or CD57: senescence) and illustrate the heterogeneity of cells involved in T cell immunity. Our ability to develop an even better understanding of T cell immune function under normal and pathological conditions will hinge on our ability to further detail immunologically relevant cell populations with additional, functionally defined markers.

We recently reported that the tetraspanin CD151 may provide such a marker and describes a subpopulation of T cells with an increased propensity to proliferate in the absence of cognate antigen exposure, solely driven by the presence of interleukin-2^22^. We now show that CD151 appears to be a sensitive T cell activation marker. Stimulation with specific antigen or anti-CD3 monoclonal antibodies triggered upregulation of CD151 on CD4 + and CD8+ T cells (Fig. 1). Under these experimental conditions, upregulation of CD151 seemed to parallel the upregulation of the established T cell activation marker CD25. However, ex vivo, in the absence of in vitro stimulation, CD25 was not present on the majority of CD151+ T cells. To explain the dissociation of these two activation markers, one could speculate that CD151 expression would be a marker of previous or recent antigen exposure. This would explain the ability of CD151+ T cells to proliferate in the continuous presence of IL-2, as IL-2 was initially described as a T cell mitogen that exclusively drives the proliferation of antigen-exposed T cells^67–69^. Consistent with its function as a T cell activation marker and reports that secondary immunological organs harbor more activated T cells than are found in the peripheral blood, CD151+ T cell frequencies were found elevated in splenocytes (Fig. 4). Interestingly, CD151+ T cells in both the peripheral blood and the spleen were largely CD25-negative, supporting the notion that CD151 and CD25 expression either have different activation thresholds or are regulated by different transcription factor signatures and possibly kinase pathways. Similarly, we could demonstrate that CD151 marks a substantial group of T cells independent of other activation markers, specifically, CD25, CD38 and PD-1 (Fig. 3e).

Studies with p38 and ERK inhibitors, a pathway that was suggested by kinome analysis to be activated in CD4+CD151+ T cells (Table 1), demonstrated that the expression of CD151 is uniquely driven by the MAP kinase pathway, with CD151 expression being sensitive to the inhibitors at baseline (Fig. 5b; Supplemental Figure S1). This effect confirms that this pathway is active in CD151+ T cells, and providing an explanation as to why IL-2 exposure of CD151+ T cells is able to drive proliferation^22^. These data, together with the dissociation of CD151 from other T cell activation markers, such as CD25, CD38 or PD-1 (Fig. 3), confirm that CD151 is a unique T cell activation marker that marks a distinct subset of T cells.

The seemingly contradicting features of CD151 expression, CD151 upregulation in response to stimulation, but high baseline expression on quiescent/senescent memory T cells, suggest different, context-dependent functions for CD151. While CD151 upregulation following T cell activation would be in line with its reported role in the stabilization of the immunological synapse^32^, its baseline expression on a percentage of naïve and quiescent/senescent memory T cells suggests that CD151 must have a function beyond supporting T cell activation. One possibility is highlighted by the expression of CD151 on almost all CD28-negative T cells, which we had previously reported and here confirm for T cells from HIV/ART patients (Fig. 6e,f). HIV/ART patients are known to have an increased pool of CD4+CD28− T cells, in particular in patients with CD4 T cell counts < 300 cells/ml when compared to healthy donors^70–74^. As loss of CD28 expression is the classic T cell senescence marker, and we have demonstrated that CD151 provides an activating outside-in signal^22^, CD151 may act to promote viability and bestow the ability to respond to TCR activation signals to these CD28- T cells. Association of CD151 expression with senescence for some T cells may further be suggested by the finding that at least a portion of the PD1+ T cells also express CD151 (Fig. 3) and that CD151+ T cells are more likely to express preformed granzyme B, another marker of senescent T cells (Fig. 6c,d). Nevertheless, it remains to be investigated what role CD151 plays on non-senescent CD28 + PD1− T cells, a subject of ongoing research in our laboratory.

Beyond the ability of CD151 to further detail populations of CD4 + and CD8+ T cells in the context of memory differentiation and in the context of the expression of other markers describing T cell states, such as CD25, CD38 or PD-1, the sensitivity of CD151 as a T cell activation marker may be clinically useful. The observed increase in the median frequency of CD4+CD151+ T cells in HIV/ART patients, and maybe more so the increase in HIV/ART patients that had CD4+CD151+ T cell counts outside the 95% reference range determined for healthy donors, may render CD151 an interesting marker of pathological immunological hyperactivation states.

Within this setting, the observed accumulation of CD151+ T cells with a hyper-responsive phenotype in memory T cell compartments could be expected, e.g. as a result of repeated antigen exposure or past improper or insufficient cognate antigen recognition^75–78^. However, the increased frequency of CD151+ T cells in the naïve T cell population may be a stronger indicator of an inflammatory environment, as reports on rheumatoid arthritis, another chronic inflammatory disease that has been associated with premature immunological aging^63,64^, describe that proliferation was most pronounced in the naïve T cell population of these patients^65^.

Taken together, the presented data suggest that CD151 will be a valuable marker to further detail our understanding of T cell populations, for both research and possibly clinical purposes.

## Methods

### Cell culture and inhibitors

For the experiments, cryopreserved PBMCs were thawed, counted and assessed for viability with a Guava EasyCyte flow cytometer (EMD Millipore, Billerica, MA, USA). Lymphocyte viability ranged from 85 to 95%. All cells were maintained in RPMI 1,640 supplemented with 2 mM l-glutamine, 100 U/mL penicillin, 100 µg/mL streptomycin and 10% heat-inactivated fetal bovine serum. Splenocytes were isolated from cadaveric human material obtained through the UAB Organ Transplant program. The utilized pharmacological inhibitors were purchased from Selleckchem (Houston, TX, USA). SCH772984 is a selective and ATP-competitive inhibitor of ERK1/2^79^. Ulixertinib is a reversible ERK1/ERK2 inhibitor with IC50 of < 0.3 nM for ERK2^80^. It is in phase I clinical trials. Losmapimod is a selective, potent, and orally active p38 MAPK inhibitor that is in phase III clinical trials^81^. Doramapimod is a pan-p38 MAPK inhibitor and was the first p38 MAPK inhibitor to be tested in a phase III clinical trial^82,83^. In all experiments, inhibitors were added to the cell cultures for 2 h before anti-CD3/CD28 mAbs stimulation and then left in the culture for the remainder of the experiments at the indicated sub-toxic concentrations (determined by initial titration for toxicity effects): Ulixertinib at 1.25 μM, SCH772984 at 0.3125 μM, Losmapimod at 10 μM, and Doramapimod at 10 μM.

### Patient descriptors

Healthy, HIV-seronegative (n = 34) and HIV-seropositive subjects (n = 52) were recruited from the 1917 Clinic cohort at the University of Alabama at Birmingham and the CFAR Network of Integrated Clinical Systems (CNICS) to donate peripheral blood. HIV-seropositive subjects were on ART and with undetectable viral loads (VL < 50 copies/mL) for a median of 10 months (6.5–17.3 months). Median age in the seronegative control group was 46 years (25–67 years) and 43 years (24–59 years) in the HIV/ART group. In accordance with the specific protocol for this project that was approved by the UAB Committee on Human Research, all subjects provided written informed consent for all biologic specimens and clinical data used in this study. Additionally, the methods performed on these studies were carried out in accordance with relevant guidelines and regulations.

### Flow cytometric analysis and reagents

LIVE/DEAD Fixable Aqua Stain or Fixable Blue Dead Cell Stain (Molecular Probes, Invitrogen, CA, USA) was used to exclude dead cells from analysis. The T cell phenotype was determined by staining with mAbs: CD3-APC efluor780 (UCHT1, eBiosciences, San Diego, CA, USA), CD4-BV785 (OKT4 or SK3, Biolegend, San Diego, CA, USA) or CD4-Qdot655 (S3.5, Invitrogen, Carlsbad, CA, USA), CD8-V500 (RPA-T8, BD Biosciences, Franklin Lake, NJ, USA), PD-1-PE-eFluor610 (J105, eBioscience), CD38-Pecy7 (HIT2, eBioscience) , CD57-Pecy7 (TB01, eBioscience), CD151-PE (14A2.H1, BD Biosciences), and CD25-FITC (M-A251, BD Biosciences) or CD25-BV421 (M-A251, BD Biosciences). Antibodies recognizing CD45RA-APC (HI100, BD Biosciences) or CD45RA-Alexa Fluor 700 (HI100, BD Biosciences) and CCR7-Percpcy5.5 (150503, BD Biosciences) were used to define naïve T cells (T_N_: CD45RA^+^ CCR7^+^), central memory T cells (T_CM_: CD45RA^–^ CCR7^+^), effector memory T cells (T_EM_: CD45RA^–^ CCR7^–^) and T effector memory RA-positive cells (T_EMRA_: CD45RA^+^ CCR7^–^) as previously reported^22^. For the detection of preformed Granzyme B, following surface staining with the above antibodies, cells were washed in PBS, followed by permeabilization with the Cytofix/cytoperm reagent (BD, San Jose, CA, USA) for 20 min at RT in the dark. Intracellular Granzyme B staining was then performed using GzmB-V450 (GB11, BD Biosciences). To determine whether protein synthesis and transport are required for upregulation of CD151 expression in PBMCs, the protein transport inhibitors monensin and brefeldin A were used to block the transport of new protein to the cell surface (BD Biosciences). Six hours after stimulation with anti-CD3/CD28 mAbs, PBMCs were cultured in the presence or absence of monensin or brefeldin A for a total of 24 h. The cells were then stained with fluorochrome-labeled mAbs: CD3-BV711 (UCHT1, BD Biosciences, Franklin Lake, NJ, USA), CD4-BV786 (SK3, BD Biosciences), CD8-V500 (RPA-T8, BD Biosciences), and CD151-PE (14A2.H1, BD Biosciences) and analyzed on a BD Symphony flow cytometer.

### Gene expression analysis

CD4+ T cells were left untreated or stimulated for 24 h with a combination of anti-CD3/CD28 mAbs. mRNA was extracted using the RNeasy Micro Kit (Qiagen, Hilden, Germany), and 75 ng of RNA was converted to cDNA using the iScript cDNA Synthesis Kit (Bio-Rad Laboratories, Hercules, CA, USA). Primer pairs (sequences were acquired from PrimerBank^84^) for CD151 and the reference genes, ATP Synthase Subunit B (ATP5PB) and TATA-Box Binding Protein (TBP) (Supplemental Table S1), were used to determine the relative expression of CD151 using qRT-PCR. Each qRT-PCR reaction, containing 10 µl of SsoAdvanced Universal SYBR Green Supermix (Bio-Rad Laboratories), 100 ng of cDNA, and 500 nM of forward and reverse primer, was run at 95 °C for 1 min, followed by 40 cycles of 95 °C for 15 s and 61 °C for 30 s. CD151 expression data were normalized using TATA-Box Binding Protein (TBP) as reference gene and relative gene expression analyses were conducted using the 2^-ΔΔCT^ method^85^.

### T cell proliferation assays

PBMC were resuspended in PBS (5 × 10^6^–10^7^ cells/ml) and labeled with 1.25 μM 5,6-carboxyflourescein diacetate succinimidyl ester (CFSE; Molecular Probes, Eugene, OR) for 4 min at room temperature with constant gentle agitation as previously reported^86^. Cells were washed twice in PBS containing 10% FBS. Finally, cells were resuspended in complete media (RPMI + 10% FBS, 50 U/ml of penicillin/streptomycin, 2 mM l-glutamine and 25 mM HEPES). Proliferation was monitored following stimulation with appropriate antigens for 4 days. For the antigen stimulation, pools of overlapping HIV peptides (15-mers overlapping by 11, NIH AIDS repository) from the Gag protein were used. Responses to CMV antigen were measured using CMV-pp65 (15-mers overlapping by 11, NIH AIDS Reagent Program). Cells stimulated with anti-CD3/CD28 mAb or SEB (Sigma-Aldrich, St. Louis, MO, USA) were used as positive controls and unstimulated cells served as negative controls. Fluorochrome-labeled mAbs: CD3-APC efluor780 (SK7, eBioscience, San Diego, CA), CD4-BV785 (OKT4 or SK3, Biolegend, San Diego, CA) or CD4-Qdot655 (S3.5, Invitrogen, Carlsbad, CA, USA), CD8-V500 (RPA-T8, BD Biosciences, Franklin Lake, NJ, USA), PD-1-PE-eFlour610 (J105, eBioscience), CD38-Pecy7 (HIT2, eBioscience) , CD57-Pecy7 (TB01, eBioscience), CD151-PE (14A2.H1, BD Biosciences), and CD25-BV421 (M-A251, BD Biosciences), CD45RA-AlexaFlour700 (HI100, BD Biosciences) and CCR7-Percpcy5.5 (150503, BD Biosciences). The cells are analyzed on a BD LSRII or a BD Symphony and at least 1 × 10^5^ CD3+ T cells were collected. Proliferation was measured as decrease in CFSE fluorescence intensity on cells in the CD3+CD4 + or CD3+CD8+ T cell gate.

### Kinome analysis

This method has been described previously^22,87^. Briefly, samples from 5 healthy donors were sorted for CD4+CD151 + and CD4+CD151− T cells. 1 × 10^6^ CD4+CD151 + and CD4+CD151− T cells from each donor were pooled into one single CD4+CD151+ T cell and one single CD4+CD151− T cell sample, processed according to the manufacturer’s instructions (Kinexus, Canada) and loaded onto the Kinexus antibody array chips. The utilized KAM-850 chips were spotted in duplicates with antibodies: 510 pan-specific antibodies used in the chip allows for the detection of 189 protein kinases, 31 protein phosphatases and 142 regulatory subunits of these enzymes and other cell signaling proteins. 340 phospho-specific antibodies tracked the unique phosphorylation of 128 sites in protein kinases, 4 sites in protein phosphatases and 155 sites in other cell signaling proteins. Each array produced a pair of 16-bit images, which are captured with a Perkin-Elmer ScanArray Reader laser array scanner (Waltham, MA, USA). Signal quantification was performed with ImaGene 8.0 from BioDiscovery (El Segundo, CA, USA) with predetermined settings for spot segmentation and background correction. The background-corrected raw intensity data were logarithmically transformed with base 2. Since Z normalization in general displays greater stability as a result of examining where each signal falls in the overall distribution of values within a given sample, as opposed to adjusting all of the signals in a sample by a single common value, Z scores were calculated by subtracting the overall average intensity of all spots within a sample from the raw intensity for each spot, and dividing it by the standard deviations (SD) of all of the measured intensities within each sample^88^. Samples with a statistically significant Z′-score were included in Table 1 (ArrayExpress accession #: E-MTAB-5977).

### Statistical analysis

Where applicable, a non-parametric Wilcoxon Signed Rank test was used for paired samples, otherwise the Mann–Whitney U test was used. Differences were considered to be significant on the basis of 95% confidence intervals (p < 0.05). For experiments determining the effect of inhibitors on CD151 expression relative to untreated cells, we used a paired, one-tailed t test. Analyses where done with GraphPad Prism software 8.0 for Mac (GraphPad Software, La Jolla, CA, USA). Differences were considered to be significant on the basis of mean and standard deviation (α < 0.05).

### Ethical approval

All research involving human samples was performed with the approval of the Institutional Review Board of the University of Alabama at Birmingham, USA.

## Supplementary information

Supplementary information
