# Supplementary material for: The tetraspanin CD151 marks a unique population of activated human T cells
Source: Sci Rep. 2020 Sep 25;10:15748. doi: 10.1038/s41598-020-72719-8 (PMC7519159; doi:10.1038/s41598-020-72719-8)
Supplement: Supplementary file 1 — Supplementary information [file 41598_2020_72719_MOESM1_ESM.pdf]

The tetraspanin CD151 marks a unique population of activated human T cells

Mildred D. Perez[1*], Lillian Seu[1*], Kelsey E. Lowman[1*], David C. Moylan[1], Christopher Tidwell[1], Shekwonya Samuel[1], Alexandra Duverger[1], Frederic H. Wagner[1], Eric Carlin[1], Vishal Sharma[1], Brandon Pope[1], Chander Raman[1], Nathan Erdmann[1], Jayme Locke[3], Hui Hu[2], Steffanie Sabbaj[1#], and Olaf Kutsch[1#]

[1]Department of Medicine, [2]Department of Microbiology, [3]Department of Surgery,

The University of Alabama at Birmingham, Birmingham, Alabama,

*contributed equally

Supplemental Figure S1

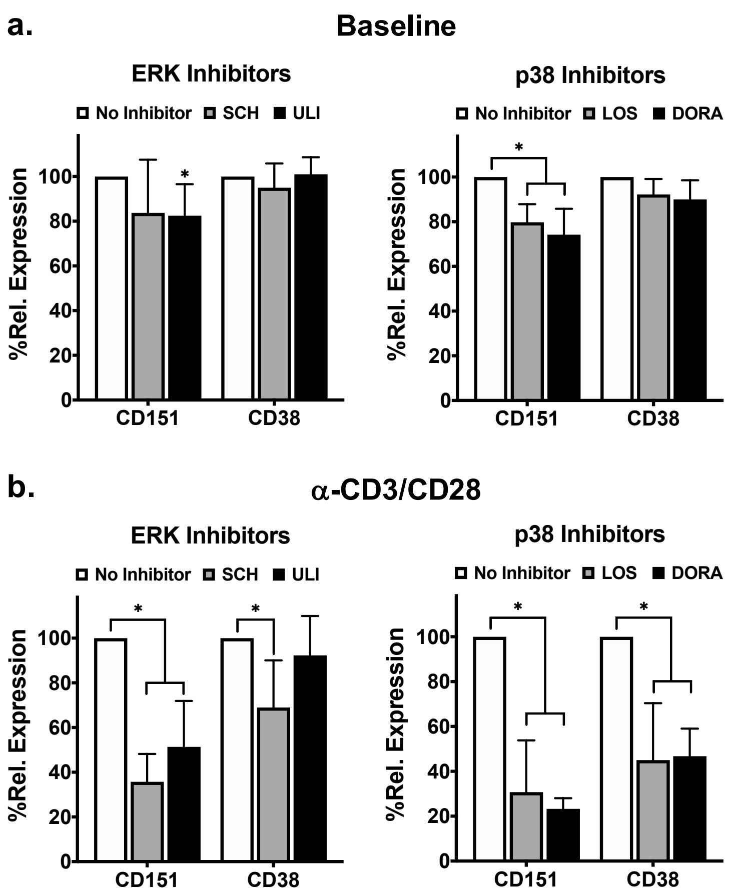

Supplemental Figure S1. Effect of ERK and p38 pathway inhibition on CD151 expression in CD8+ T cells. PBMCs from four healthy individuals were treated with the ERK inhibitors (Ulixertinib, ULI or SCH772984, SCH), the p38 inhibitors (Losmapimod, LOS or Doramapimod, DORA), or DMSO as control, and 3 days post treatment analyzed for the expression of CD151 and CD38 using flow cytometric analysis. The effect of each inhibitor on (a) baseline or (b) activation-induced CD151 expression levels in comparison to the inhibitor effect on CD38 expression as determined for four individuals. Due to extensive donor variation regarding CD151 expression levels data are represented as relative MFI normalized for the CD151 baseline expression in untreated cells.

Supplemental Figure S2

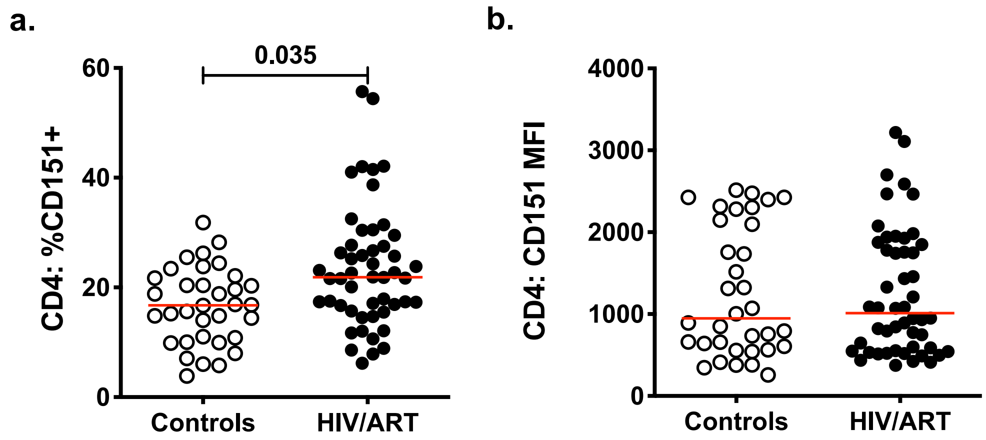

Supplemental Figure S2. CD4+CD151+ T cell frequency and CD151 expression in the peripheral blood of HIV-1 patients on fully suppressive ART. Beeswarm plots describing the (a) frequencies or (b) expression of CD4+CD151+ T cells from healthy donors (controls), and HIV-seropositive individuals on ART (HIV/ART). Red lines indicate median frequencies.

Supplemental Table S1: Table of primers used in qRT-PCR experiment.

Gene Name	PrimerBank ID	Sequence (5' → 3')	T_m (°C)	Amplicon Size (bp)
Target Gene				
CD151	87159810c1	f: ATGGGTGAGTTCAACGAGAAGA	61.0	154
		r: GCAGGCTGATGTAGTCACTCT	61.3	
Reference Genes				
ATP5PB	85794838c1	f: AGGTCCAGGGGTATTGCAG	60.7	117
		r: TCCTCAGGGATCAGTCCATAAC	60.4	
TBP	285026518c1	f: CCACTCACAGACTCTCACAAC	60.0	127
		r: CTGCGGTACAATCCCAGAACT	61.8	
